# Response to intervention as an identification strategy of the risk for dyslexia

**DOI:** 10.1590/2317-1782/20242023031en

**Published:** 2024-06-10

**Authors:** Mariana Gobbo Medda, Thais Barbosa, Isadora Salvador Rocco, Claudia Berlim de Mello

**Affiliations:** 1 Departamento de Psicobiologia, Universidade Federal de São Paulo – UNIFESP - São Paulo (SP), Brasil.

**Keywords:** Dyslexia, Specific Learning Disorder, Intervention, Learning, Learning Problems

## Abstract

**Purpose:**

To develop on intervention process to identify children at risk of dyslexia, based on the Response to Intervention model. Specifically, to identify the pattern of changes in post-intervention performance in tasks of phonological awareness, working memory, lexical access, reading and writing; and to analyze which cognitive functions had a significant effect on the discriminating students at risk of dyslexia.

**Method:**

Sample of 30 participants with Reading and writing difficulties, aged 8-11, from public/private schools, students from 3rd to 5th grade. Participants were submitted to a battery of cognitive-linguistic tests, before and after 12 intervention sessions. To monitor their performance, five reading and writing lists of words and pseudowords were applied. We qualitatively and quantitatively analyzed the differences in pre- and post-intervention performance of each participant; and among participants in the post-assessment, to understand the patterns of dyslexia vs non-dyslexia groups.

**Results:**

There were statistically significant changes in: rapid automatized naming, narrative text comprehension, phonological awareness, rate and typology of hits/misses in reading and writing, and reading speed. Being the last three variables the most sensitive to discriminate the two groups, all with less post-intervention gains for the dyslexia group.

**Conclusions:**

The intervention focused on the stimulation of phonological skills and explicit and systematic teaching of graphophonemic correspondences contributed positively to the evolution of the group's participants. The intervention response approach favored the identification of children with a profile at risk for dyslexia, as distinct from children with other learning difficulties.

## INTRODUCTION

A specific learning disorder with reading impairment - Dyslexia - is the most common type of learning disorder. The Diagnostic and Statistical Manual of Mental Disorders - DSM 5^(1)^ defines dyslexia as a condition of neurobiological origin, characterized by a pattern of persistent difficulties in decoding, spelling, and reading fluency. Thus, it differs from learning difficulties (LD) in reading and writing from extrinsic factors such as unfavorable socioeconomic conditions, educational inefficiency, lack of adequate stimulation in the family environment, emotional-affective factors, or secondary to the presence of other diagnoses.

Given the heterogeneity of the causes of LD and recognizing the diagnostic limitations, studies^(2,3)^ highlight the relevance of prevention models associated with the early identification of signs of risk for dyslexia. These models are called Response to Intervention (RTI), which generally presuppose a set of evaluative and remedial processes. Despite the variations, traditional RTI is applied in schools, in which the three-tier model is most used. These tiers mean the different implementation phases, characterized by specific and increasingly intensive intervention focus. The student is assessed for the current acquisitions in reading and writing, intervention, and reassessed with performance continuously monitored to observe changes in the learning rate. At the end of each tier, the absence of improvement in performance or slow and insufficient evolution may indicate a low response to the intervention. The persistence of this pattern is one of the diagnostic requirements for dyslexia^(3)^.

Intervention models based on phonological processing have been the most recommended for children at risk for dyslexia since difficulties in phonological awareness, verbal working memory, and lexical access speed are identified as the symptomatic triad^(4)^. There is evidence that the systematic and explicit stimulation of phonological skills combined with instruction in grapheme-phoneme correspondence helps the understanding of the alphabetic principle, as well as the adequate development of metaphonological skills, favoring the acquisition of reading^(5-7)^. One of the methods for teaching reading and writing is phonics, which stimulates phonological awareness and multisensory stimulation^(8)^. Multisensory stimulation combines different sensory modalities to encourage the child to establish connections between visual (word spelling), auditory (phonology), tactile (tactile memory of the shape of letters/words), and synesthetic aspects of spelling (perception of the coordinates needed to write) and articulation (movements to pronounce sounds consciously and intentionally).

However, in developing countries like Brazil, the implementation of RTI programs has received little emphasis, despite the initiatives of researchers^(3,9)^. In public schools, the high number of students per class is a challenge for teachers in adopting individualized pedagogical strategies. Thus, the recurring procedure to deal with the alarming number of students with low academic performance has been referral to specialized diagnostic services that carry out specific assessments, which generally involve formal tests of cognitive abilities and academic achievements based on chronological age and school level. A consequence of assessments of this nature may be the occurrence of false-positive students with dyslexia.

Therefore, early interventions based on cognitive-linguistic stimulation, performance monitoring, and sharing of pedagogical challenges among professionals are essential characteristics of RTI. One of the potential benefits is to avoid the high number of referrals for diagnostic evaluation by health professionals, in a clinical context, before a pedagogical approach in a school environment. Although indicated in specific situations, such evaluation processes should consider how individuals respond to previous intervention, incorporating multidisciplinary evaluation and intervention strategies^(2)^. This is a perspective in line with the DSM-5^(1)^ guidelines for diagnosing dyslexia, which presuppose the persistence of signs of risk or specific difficulties after systematic intervention.

Models of this nature can also contribute to the refinement of diagnostic processes in a clinical context. In other words, an approach integrating assessment and intervention actions can favor discrimination between learning difficulties and a profile suggestive of a specific disorder such as dyslexia, which is fundamental for pedagogical adequacy.

From this perspective, this study aimed to develop an intervention process to identify children at risk for dyslexia based on the intervention response model for students in Elementary School. The process prioritized one of the basic assumptions of RTI, which is to stimulate cognitive-linguistics skills in children with school difficulties and check how they respond to strategies, before diagnosis. The program was structured in stages and involved assessment and intervention processes focusing on cognitive-linguistic skills as tier 2 of the original RTI. By observing how children respond to the strategies taught, it is better to refer to the interventions, whether in a therapeutic or pedagogical context. In other words, although based on fundamental parameters, the proposal does not imply a direct relationship with the original RTI model, applied in schools. On the other hand, the program is organized in a multidisciplinary approach as it demands a coordination of actions between education and health professionals, such as speech-language therapists and psychologists.

## METHOD

The study design was analytical, observational, and cross-sectional, with repeated measures. The regulatory guidelines for research with human beings followed Resolution 466/12 of the National Health Council. The project was previously approved by the Research Ethics Committee (opinion 134234/2018). The children and their guardians were informed about the purposes and procedures of the study, so they signed the Informed Consent Form (ICF) and Assent Forms before data collection.

### Participants

The study included 30 children with a reading and writing performance profile at risk for dyslexia, between 8 and 11 years old, from the 3rd to 5th grade of Elementary School in public and private schools in São Paulo. All of them were recruited from those referred for evaluation at the Children's Neuropsychological Care Center (NANI- *Núcleo de Atendimento Neuropsicológico Infantil*), due to complaints of school difficulties. NANI is one of the units of the *Centro Paulista de Neuropsychologia*, an institute supported by the Research Incentive Fund Association (AFIP- *Núcleo de Atendimento Neuropsicológico Infantil*) and academically linked to the Department of Psychobiology of the Federal University of São Paulo/UNIFESP, developing teaching and research actions focused on the multidisciplinary approach to neurodevelopmental disorders. The search for diagnostic evaluation at NANI generally occurs through referral from medical services or educators, including children whose families spontaneously sought diagnostic evaluation or were referred by the school, considering learning difficulties as their main complaints.

The risk indicators for dyslexia were based on previous history data: (a) presence of difficulties since the beginning of school as documented in school reports and statements from guardians and (b) having participated in different activities focused on reading and writing in the school environment; and in data from the assessment of cognitive-linguistic skills: (c) lower performance in standardized tests of phonological awareness, verbal working memory, speed of lexical access, reading and writing of words, pseudowords and text and (d) evidence of an atypical pattern of errors in reading and writing tests.

The exclusion criteria considered a history of pre-, peri- or post-natal complications with a risk of neurological changes such as birth before the 36th week of gestation; already established diagnosis of autism and/or dyslexia (informed by the family), intellectual disability (IQ < 70) or neurological, genetic or psychiatric diseases; presence of uncorrected changes in visual or hearing acuity. Children with signs of risk for attention deficit hyperactivity or language disorder were not excluded from the sample given the high prevalence of comorbidity with dyslexia. None of the children selected for the study regularly used medications that act on the central nervous system. The identification of risk signs for the different neurodevelopmental conditions occurred after the pre-intervention assessment (the initial process of the study).

### Procedures

A total of 68 children were initially considered eligible for the study based on prior history indicators and underwent pre-intervention assessment. Among them, 38 did not meet inclusion criteria based on performance in cognitive-linguistic tests and were not included in the study, although they continued the multidisciplinary assessment at NANI. Therefore, a final sample of 30 children was subjected to the intervention process.

### Characterization of the evaluation-intervention program

**Figure 1:**
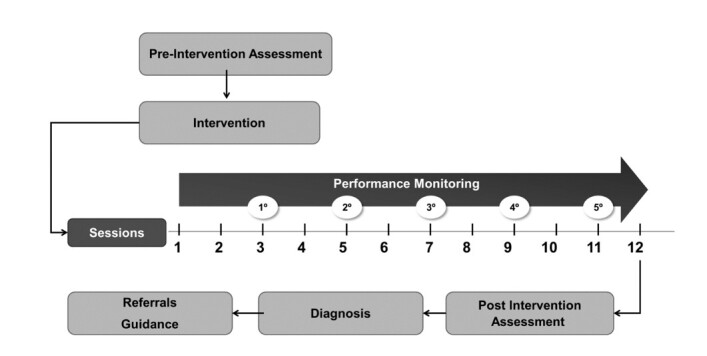
General scheme of the study stages

Unlike the traditional model (that is, carried out in a school context and at three tiers of intervention), the program was designed based on the parameters of Tier 2 of the RTI. Three stages were stipulated from both an evaluative and remedial perspective^(1)^: pre-intervention assessment^(2)^; intervention and performance monitoring; and^(3)^ post-intervention assessment (Figure 1). All stages were conducted by specialists in neuropsychology, speech-language therapy, and/or psychopedagogy.

### Pre and post-intervention assessment

In an initial interview, those responsible provided information on the neurodevelopmental, clinical, and educational history, and responded to the Brazilian version of the Child Behavior Checklist - CBCL^(10)^. Subsequently, the children were submitted to the Wechsler Abbreviated Scale of Intelligence-WASI^(11)^. These procedures specifically aimed to meet the adopted exclusion criteria. The pre- and post-intervention assessment included the investigation of cognitive-linguistic skills using instruments developed for this purpose, as follows. The Word and Pseudoword Repetition Tests^(12)^, digit span tasks, and Corsi-Block span tasks (repetition in reverse order)^(13)^ were used to assess working memory. Lexical access speed was analyzed using the Rapid Automatic Naming Test for objects, colors, numbers, and letters, that is, Rapid Assessment Naming-RAN^(14)^. Syllabic and phonemic awareness skills were assessed using the Phonological Awareness by Oral Production test^(15)^. In the assessment of reading and writing skills, the Reading Process Assessment Tests-PROLEC^(16)^, Word Reading and Pseudoword subtests were used, considering the rate of correct answers for words (low and high frequency) and pseudowords, as well as the error rate (total number of phonemes in each stimulus). The speed and comprehension of reading aloud were assessed based on two texts from the Text Comprehension Subtest-PROLEC^(16)^. The assessment focused on the semantic process (percentage of questions answered correctly and the number of words read per minute). To assess writing, the correct answers, error rate, and error typology of the Writing under Dictation test were analyzed^(17)^. Errors in reading and writing were categorized according to specific typology^(18)^: lexicalization, substitution of voiceless phonemes, inversions, omissions and/or additions, lack of knowledge of contextual rules and spelling, exchange of vowels, and other errors. Finally, to evaluate the narrative structure, the child was asked to write a story based on a sequence of images. The analysis followed categorical criteria suggested in studies by Spinillo and collaborators^(19)^, adapted for this study: non-response (refusal to write), non-story (writing of poorly connected sentences), descriptive production (without the construction of a sequence of events or causal relationships), complete story (elaborate narrative structure, which may have more restricted vocabulary and short production, as long as it presents a beginning, middle and end, presentation of the conflict and outcome).

### Intervention

The 30 participants were allocated to groups of five children, as interventions and response observations would be more effective in smaller groups. The grouping considered the chronological age and school schedule of each participant since the interventions took place after school. Other criteria were considered, when possible, such as the level of performance in reading and writing, which in the sample ranged from more basic acquisitions, such as recognition of isolated words, to an elementary ability to understand texts. It is important to mention that a strict grouping according to reading level proved to be unfeasible given the heterogeneity of the sample's performance but it reflects the reality in classrooms.

The intervention was designed as a structured and systematic approach to stimulating reading and writing coding and decoding skills. The stimulation was multisensory, with the initial objective of providing basic strategies for discriminating between phonemes, and teaching their auditory and articulatory characteristics, necessary for children to later be able to represent sequences of sounds in syllables. The tasks followed an order of complexity, from the most basic concepts to the most complex. Concrete materials, such as cardboard, movable letters, and mirrors, were used as facilitators. Oral activities were always aligned with associations with figures from different categories. Participants were frequently encouraged to reflect on their responses and performance, becoming able to self-correct.

The intervention was structured into 12 two-hour sessions, one each week. The frequency of the sessions was due to the possibility of families to attend the service. Both the organization of the sessions and the tasks taught were based on and adapted from studies with the applicability of RTI in schools, as in Andrade and Capellini^(3)^, Almeida and collaborators^(9)^; and in other intervention programs such as those of Seabra, Capovilla^(8)^.

In the first six sessions, the focus was on the explicit and systematic teaching of more basic phoneme-grapheme correspondence skills through multisensory stimulation, using phono-visuoarticulatory strategies and phonic stimulation. At this stage, aspects of oral language were studied such as phonological awareness, phoneme discrimination (focusing on voiceless-deaf pairs, providing articulatory tips), auditory/verbal memory, such as repetition and manipulation of verbal information, categorization, naming, and vocabulary. The proposal was to explore the semantic characteristics of words and their use in simple sentences, moving on to complex ones and the use of specific vocabulary in structured oral narratives. In the following six sessions, emphasis was placed on more complex elements, focused on writing, and reading sentences and texts. Reading was encouraged mainly aloud and shared, with less demand for silent and individual reading. Strategies were used to stimulate the phonological route, with explicit teaching of correspondences, and lexical, for example, with lists of words that share orthographic rules and their exceptions, also stimulating the visual memory of the words. We tried to intersperse tasks with an emphasis on stimulating cognitive-linguistic skills, such as word reading or working memory, with more playful, short, and shared activities, such as games, or short breaks to minimize tiredness or loss of engagement.

### Monitoring performance throughout the intervention

To monitor the participants' performance, data were collected from reading and writing assessments of five lists of words and pseudowords, administered every fortnight, in an individual session. The lists were composed of 15 items selected from Pinheiro's study^(20)^, five high-frequency words, five low-frequency words, and five pseudowords. The stimuli varied with each test, maintaining psycholinguistic characteristics regarding regularization, lexicalization, and frequency. The monitoring aimed to analyze each child's learning curve, considering accuracy (hit rate, error rate, and typology of errors in reading and writing) and reading speed.

### Data analysis

Data analysis was conducted in three stages. The first involved a repeated measures mixed model approach (Generalized Mixed Model - GMM) and was conducted using the IBM SPSS 21 program. For each variable, the effect of time was tested (comparison analysis between A1 - pre-intervention and A2 - post-intervention) and five times analysis (five occurrences of performance monitoring evaluation, L1 to L5), to identify variables with significant changes, considering the entire sample (n=30). Therefore, we had a total of seven assessment moments (pre and post-intervention and five monitoring assessments). The second stage focused on defining responsiveness to the intervention. Individual variation rates (delta rate) were calculated to identify how much everyone changed in A2 according to their performance in the pre-intervention phase (only variables at a significant level of p<.05 in the pre and post-comparison of the entire sample were analyzed). These values were organized into median and interquartile ranges. Thus, participants who were unable to exceed the lower limit (i.e., 25% quartile) in the rate of variation showed a low response to the intervention. We consider the 25th percentile as an indicator of low responsiveness, according to clinical indicators based on psychometric properties^(21)^. We also considered the analysis of the percentage curve of errors and correct answers in reading and writing words and pseudowords in the monitoring stage and the typology of errors, and qualitative analyses of performance. Based on the response analysis to the intervention in this stage, the 30 participants were classified into two groups: participants with a risk profile for dyslexia (GD n=12) versus non-dyslexia (GND n=18). To analyze participants' responsiveness and group discrimination, the variables were considered (a) phonological awareness, (b) lexical access, (c) rate of errors and successes in reading/writing, (d) reading time and accuracy, (e) typology of errors in reading and writing. Finally, in the third stage, a generalized model (Generalized Linear Model statistical test - GLzM), controlled by age (covariable), was conducted to verify possible differences between the groups considered in the post-intervention situation. The prerequisites were tested for all dependent variables and significance was found at the general level of p<.05.

## RESULTS

According to the sociodemographic characterization of the sample, the average age was 9.2 years (SD=.94), with a predominance of boys (60%) and public-school students (66.7%), with 36.7% students from the 3^rd^ grade, 33.3% from the 4^th^ and 30% from the 5^th^ grade.

### Comparison of pre- and post-intervention measures

Tables 1 and 2 show the data referring to Stage 1 of data analysis and express differences in performance in the domains of working memory, lexical access, phonological awareness, reading, and writing between the pre- and post-intervention assessments, in the sample (n=30). Statistically significant differences were observed in the lexical access tests, that is, naming objects (F=5.66/p=.024), numbers (F=5.21/p=.030), and letters (F=10. 02/p=.004); phonological awareness (F=35.16; p<.001), in the syllabic domains, in syllabic manipulation (F=8.76/p=.005) and alliteration (F=4.60/p=.040); and phonemic in synthesis (F=9.05/p=.005); segmentation (F=19.33/p<.001) and manipulation (F=4.79/p=.037), with improvement in A2, as shown in Table 1.

**Table 1:** Difference in performance of the total sample in the pre- and post-intervention assessment for the domains: Operational Memory; Lexical Access; Phonological Awareness

**Domain Variable** a				**Mean (Standard-Deviation)**		**Confidence interval 95%**		**Estimates** b	**t**	**Sig.**
				**Pre**	**Post**	**Pre**	**Post**			
Operating memory		Digits		49.10(2.0)	50.00(1.6)	45.0-53.1	46.5-53.4	0.90	-.365	.718
		Corsi		48.80(1.8)	52.83(1.8)	45.0-52.5	49.1-56.5	4.03	-1.96	.059
		WRT		49.05(2.5)	53.00(2.6)	43.6-54.4	47.5-58.4	3.95	-2.05	.054
		PRT		50.81(1.4)	49.05(1.8)	47.8-53.7	45.1-52.9	-1.76	1.04	.310
Lexical Access	RAN objects			39.80(1.4)	42.80(1.4)	36.9-42.6	39.9-45.6	3.00	2.38	.024*
	RAN colors			39.53(1.4)	39.13(1.5)	36.5-42.5	36.0-42.2	-.40	-.34	.734
	RAN numbers			38.53(1.1)	40.66(1.1)	36.1-40.9	38.2-43.0	2.13	2.28	.030*
	RAN letters			36.10(1.4)	39.70(1.4)	33.2-38.9	36.8-42.5	3.60	3.16	.004*
Phonological Awareness	Syllabic		Synthesis	47.07(2.2)	50.20(1.3)	42.4-51.6	47.5-52.8	3.13	1.39	.175
			Segmentation	52.10(.98)	52.93(.98)	50.1-54.0	50.9-54.9	-.83	-.71	.478
			Manipulation	49.23(1.7)	55.20(1.0)	45.7-52.7	53.0-57.3	5.96	2.96	.005*
			Transposition	49.13(1.5)	51.50(1.4)	46.0-52.2	48.5-54.5	2.36	1.32	.197
			Rime	40.47(2.5)	43.73(2.4)	35.2-45.6	38.7-48.7	3.26	1.40	.172
			Alliteration	40.50(2.6)	47.13(2.6)	35.1–45.8	41.7-52.4	6.63	2.14	.040*
	Phonemics		Synthesis	38.86(1.4)	45.70(2.0)	35.9-41.8	41.5- 49.8	6.83	3.00	.005*
			Segmentation	43.30(1.5)	51.00(1.7)	40.1–46.4	47.5-54.4	7.70	4.39	<.001*
			Manipulation	40.06(2.0)	44.50(2.0)	35.9-44.1	40.3-48.6	4.43	2.18	.037*
			Transposition	42.80(1.3)	45.76(2.0)	40.1-45.4	41.6-49.8	2.96	1.65	.109
	P.A. Total			37.13(2.2)	46.60(2.30)	32.4-41.8	41.8-51.3	9.46	5.93	<.001*

Generalized Mixed Model Statistical Test (GMM)

a Dependent variable: Digits and Corsi - *Reverse order span*

b Mean Difference

* Significant difference at p<0.05

**Caption:** WRT= Word Repetition Test – total hits; PRT= Pseudoword Repetition Test – total hits; RAN= rapid automatic naming – total appointment time in seconds; P.A. Total= Phonological awareness total hits

**Table 2:** Difference in performance of the total sample in the pre- and post-intervention assessment for the Reading and Writing domains

**Domain**		**Variable** a	**Mean (Standard-Deviation)**		**Confidence interval 95%**		**Estimates** b	**t**	**Sig.**
			**Pre**	**Post**	**Pre**	**Post**			
Reading	Correct	Total	52.29(4.7)	69.23 (4.7)	42.5-62.0	59.5-78.9	16.94	6.02	.001*
		HF word	30.20(2.6)	38.26 (2.6)	24.8–35.5	32.9–43.6	8.06	3.48	.002*
		LW word	23.03(1.2)	29.33(2.3)	20.4-25.6	24.5-34.1	6.30	2.88	.007*
		PP	21.53(1.3)	25.16(1.3)	18.8-24.1	22.5-27.8	3.63	2.67	.012*
	Errors	Total	24.34(4.5)	9.97(4.5)	15.1-33.5	.74 -19.2	-14.37	-3.20	.003*
	Nar. text	WPM	46.89(5.2)	57.67(5.0)	36.1-57.6	47.3-68.0	10.77	3.48	.003*
		TC	48.29(7.5)	71.87(7.1)	32.9-63.6	57.1-86.5	23.58	3.77	.001*
	Exp .text	WPM	36.22(4.7)	45.18(4.6)	26.5-45.8	35.6-54.6	8.96	3.81	.001*
		TC	72.65(5.4)	69.79(6.2)	60.4-84.8	56.9-82.6	-2.86	-.587	.569
Writing	Correct	Total	29.73(4.5)	41.10(4.5)	20.4-38.9	31.8-50.3	11.36	5.92	<.001*
		Pal.	30.10(4.7)	41.36(4.3)	20.2-39.9	32.4-50.3	11.26	4.96	<.001*
		PP.	28.16(4.8)	40.80(4.8)	18.3-37.9	30.9-50.6	12.63	4.27	<.001*
	Errors	Total	35.53(5.0)	21.66(5.0)	25.2-45.7	11.4-31.9	-13.86	-3.62	.001*
		Pal.	27.76(2.1)	30.06(2.1)	23.3-32.2	25.6-34.5	2.30	2.56	.016
		PP.	30.06(2.6)	34.46(2.6)	24.7-35.3	29.1-39.7	4.40	2.97	<.001*

Generalized Mixed Model (GMM) statistical test

* mean difference is significant at the .05 level

a Dependent variable: Reading Correct Answers (Total= % correct total score);

b Mean Difference

**Caption:** HF= high-frequency word, LF= low-frequency word); PP= pseudoword - PROLEC test, standard score); Reading Errors (Total = % error total score). Narrative text (NAR); Expository text (EXP); WPM= words per minute; TC= text comprehension: % of correct questions. Writing Total Hits, Words (Pal), Pseudoword (PP): values referring to the average % of correct answers; Writing Errors: Total: average % of errors. Pal. and PP - % of the standard score

There were differences between the reading and writing rates of words and pseudowords obtained between A1 and A2 (Table 2). In reading, there was an increase of 16.94 points on average in the rate of total correct answers (F=36.31/p<.001); in hit rates in high-frequency words (F=12.14/p=.002), low-frequency words (F=8.34/p=.007) and pseudowords (F=7.16/p=.012). The error rate decreased by 14.37 points on average (F=10.27/p=.003). There was a difference in the number of words read per minute (PPM) in narrative text (F=12.17/p=.003) and expository text (F=14.54/p=.001). These advances seem to have favored reading comprehension of the narrative text (F=14.49/p=.001), given the increase in the number of correct answers in the questionnaires. Writing increased by 11.36 points on average in the correct rate (F=35.09/p<.001), with a gain of 11.26 points on average in words (F=24.59/p=<. 001) and 12.63 points on average in pseudowords (F=18.23/p=<.001). The total error rate decreased significantly (F=13.14/p=.001), in words (F=6.57/p=.016), and in pseudowords (F=8.85/p<.001), participants gained scores in the standard test. The writing task progressed with transition from one category to another (F=11.9/p=.002), for example, in A1, 33.3% of the sample performed in the initial /non-responses/ category already in A2, the predominance was 60% of /complete productions/, representing a 50% increase in the total frequency for this category. In A1, 30% of the sample produced /descriptive/ stories, and in A2, 6.7% remained in this category.

### Performance monitoring

There was a significant effect of time on the total correct reading rate (F=4.47/p=.003), especially among the first three lists (L1, L2, L3); in pseudowords (F=5.55/p=.001) and in the accuracy index (F=2.73/p=.035). There was no significant change in the reading of high (F=.508/p>.05) and low-frequency words (F=1.49/p>.05). There was a decrease in the total error rate (F=7.68/p<.001) between the first two lists compared to the others; in high-frequency words (F=2.66/p=.040), low frequency (F=4.95/p=.002) and in pseudowords (F=10.20/p<.001). In writing, there was no increase in the rate of total correct answers, but when the stimuli were analyzed separately, we identified changes in the writing of low-frequency words (F=8.19/p<.001); of high frequency (F=3.76/p=.008) and in pseudowords (F=3.49/p=.012). The decrease in the total error rate (F=2.87/p=.032) when comparing L1 with L5 (p=.012) can reinforce the gains obtained in the post-evaluation, in which an increase in the test score was noted (indicating a reduction in errors). Analysis of the stimuli showed a significant difference in the error rate for low-frequency words (F=3.19/p=.024), high-frequency words (F=11.02/p<.001), and pseudowords (F=7.75 /p<.001).

The analysis of the typology of errors for reading in the seven times (A1, A2 and five monitoring lists) showed a reduction in errors due to the substitution of voiceless phonemes (F=13.13/p<.001); omission and/or addition of letters (F=3.28/p=.006); lack of knowledge of spelling rules (F=14.05/p<.001); vowel change (F=5.99/p<.001); lexicalization (F=5.96/p<.001); others (F=2.56/p=.022). In writing, there was a reduction in errors due to deaf-sound exchanges (F=6.85/p<.001); inversion (F=3.15/p=.007); omission and/or addition (F=6.63/p<.001); spelling (F=20.35/p<.001); vowel change (F=6.63/p<.001); and others (F=4.11/p<.001).

### Characterization of the profile of the Risk Group for Dyslexia (DG) and the Non-dyslexia Group (NDG)

Chart 1 shows the association of qualitative analysis criteria established for the identification of DG. The table shows the retrospective analysis of the intervention sessions based on individual observation records and corroborates the quantitative results obtained in statistical tests since no single criterion is sufficient to configure the dyslexia profile.

**Chart 1:** Criteria for identifying profile groups at risk for Dyslexia vs Non-dyslexia

Pre-Post Assessment and Monitoring	Dyslexia	Non-dyslexia
Phonological awareness	< rate of change of gain in score	> rate of change, of gain in score
Lexical Access	< rate of change of gain in score	> rate of change, of gain in score
Error and correct answers rate	Oscillating or stable learning curve, with little change	Gradual reduction in errors
	< rate of change of gain in score	Increase in hits
		> rate of change, of gain in score
Error typology	Specific pattern in reading and writing. Maintenance of errors by substitution of deaf-sound phonemic pairs.	Varied pattern
Reading speed	Slow Evolution, low change	Varied
**Session criteria**	**Dyslexia**	**Non-dyslexia**
Phonological awareness	Maintenance of difficulty (or advances and setbacks) in tasks of manipulating words in the sentence (identification, word replacement); manipulation with syllables; separation and joining of words in oral sentences; manipulation of rhymes, identification and manipulation of phonemes.	Difficulty in the first sessions and a reduction in errors and/or difficulties in carrying out tasks, more evident from the 6th session onwards.
Phonovisuoarticulatory stimulation	Maintenance of difficulty in oral activities of discriminating phonemic pairs; need for multiple repetitions; slowness to automate sound discrimination strategies.	Difficulty in the first sessions, with gradual decrease; greater understanding and identification of peer differences; the faster process of discrimination and maintenance of strategies.
Reading and writing	Maintenance of errors due to deaf-sound exchanges, more evident after the 6th session; slow evolution in self-correction capacity, need for constant direction.	Reduction of errors due to deaf-sound exchanges, more evident from the 6th session onwards; evolution in the ability to self-correct; spontaneous resumption of taught discrimination strategies.
	Slow evolution in phoneme-grapheme correspondence (sequencing and spelling).	Maintenance of spelling, regularization, and contextual errors.
	Maintenance of errors due to hypo/hypersegmentation, little self-correction.	
	Maintains spelling, regularization, and contextual errors.	
General aspects (memory and access)	Slowness in naming figures, often referring to “thing”, or “business” to name images even from common categories (everyday objects, food, animals)	Varied pattern.
	Maintenance of difficulties in mental manipulation.	No difficulty or slowness was observed in naming pictures, except for the vocabulary repertoire being reduced.
		Maintenance of difficulties in mental manipulation.

**Figure 2:**
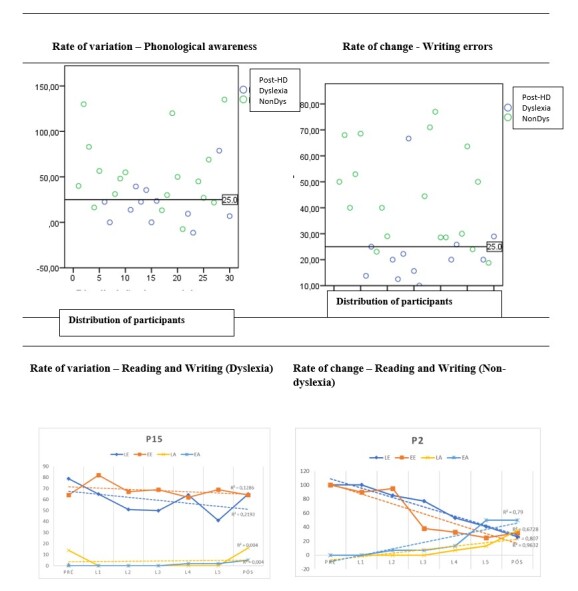
Change profile and comparison between groups

The analysis of the individual error/correctness curve in reading/writing identified patterns that were also analyzed to discriminate the groups. Figure 2 exemplifies the performance of two participants in the reading/writing tasks. Inspection of the comparison data between A1 and A2 showed that participants with performance below 25% in the individual variation rate were considered low responders, compared to the others in the group.

Y-axis: percentage of errors and/or correct answers in reading and writing tasks. Axis x: Av. pre-intervention, Monitoring List 1, List 2, List 3, List 4, List 5, Post-intervention evaluation. Measures evaluated: LE (reading errors); EE (writing errors); LA (reading correct); EA (writing successes). P15-GD patient. P2-GND patient

The association of criteria indicated 12 children with a risk profile for dyslexia. The comparison analysis showed a significant effect of group on the rate of variation in phonological awareness (Wald=6.074; p=.014); errors in reading (Wald=4.103; p=.040) and writing (Wald=4.367; p=.026); expository text reading speed (Wald=4.572; p=.032). The DG presented lower values on average regarding the gain achieved in these measures compared to the NDG (Table 3). Regarding the typology of errors in reading, the DG had more errors due to deaf-sound exchanges (F=4.66; p=.040), while the NDG had more errors due to lack of knowledge of spelling rules (F=5.72; p=.021). The comparison between means and frequency indicated that although the DG had more errors due to lexicalization and vowel changes, the difference was not reliable at p<0.05. In writing, DG made more errors due to deaf-sound exchange (F=9.52/p=.019), while NDG had more errors due to omission and/or addition of letters (F=4.08/p=.040). The comparison between means indicated that errors due to vowel changes were more committed in the DG, but the difference was not reliable at p<0.05.

**Table 3:** Comparison of individual variations by group

**Domain**	**HD**	**M (DP)**	**Confidence interval 95%**		**B** ^a^	**Hypothesis test**	
			**Lower**	**Upper**		**Wald Chi-Square**	**Sig.**
TPA	DG	11.63 (10.0)	-8.00	31.28	-33.60	6.07	.014*
	NDG	45.24 (7.9)	29.64	60.83			
RAN (Letters)	DG	8.10 (7.0)	-5.79	22.00	-8.81	.83	.361
	NDG	16.92 (5.6)	5.88	27.95			
RAN (Obj.)	DG	11.14 (6.0)	-.69	22.97	3.06	.13	.709
	NDG	8.08 (4.7)	-1.31	17.47			
RAN (Num)	DG	6.11 (4.3)	-2.41	14.63	-1.16	.04	.836
	NDG	7.27 (3.5)	.308	14.23			
VOM	DG	-.39 (10.8)	-21.57	20.78	-14.21	1.03	.308
	NDG	13.82 (8.8)	-3.46	31.11			
MOW	DG	6.06 (4.3)	-2.50	14.63	2.98	.266	.606
	NDG	3.08 (3.78)	-4.33	10.50			
COM	DG	7.60 (8.1)	-8.30	23.51	-8.44	.586	.444
	NDG	16.05 (6.4)	3.42	28.67			
Reading ER	DG	38.46 (6.5)	25.56	51.36	-17.44	4.21	.040*
	NDG	55.91 (5.3)	45.38	66.44			
Reading CA	DG	10.62 (4.4)	10.82	19.42	-10.51	2.96	.085
	NDG	21.13 (3.5)	14.15	28.11			
Writing ER	DG	23.38 (5.7)	12.13	34.62	-16.48	4.95	.026*
	NDG	39.86 (4.6)	30.68	49.04			
Writing CA	DG	9.56 (3.0)	3.67	15.44	-3.01	.54	.461
	NDG	12.57 (2.3)	7.89	17.24			
PPM NAR	DG	15.38 (14.0)	-12.08	42.84	-26.25	1.79	.180
	NDG	41.63 (12.3)	17.33	65.93			
PPM EXP	DG	13.87 (12.1)	-9.87	37.62	-35.90	4.57	.032*
	NDG	49.77 (10.8)	28.42	71.12			

a Difference between the average variation rate DG x NDG. Reference RG: DG

* Significant difference at p<0.05

**Caption:** variables: TPA (Total phonological awareness); Lexical access (rapid automatic naming (RAN) of letters, objects, numbers); VOM (digit verbal operational memory); MOW (memory operational words); COM (Corsi visual operational memory); ER reading (error); CA reading (correct answer); Writing ER (error); CA writing (correct); PPM NAR (reading speed word per minute narrative text); PPM EXP (reading speed word per minute expository text). The model covariates are set at the following values: AGE=9.27

## DISCUSSION

This study developed and analyzed the results of a program to detect signs of risk for dyslexia involving intervention strategies focusing on cognitive-linguistic skills and graphophonemic correspondence training, as proposed in Tier 2 of the Response to Intervention model carried out in a school context. To this end, a sample of 30 children from Elementary School were subjected to intervention activities, with performance monitoring and pre- and post-intervention evaluation.

In the post-intervention assessment, we initially analyzed changes in performance in the sample as a whole and then the significant cognitive-linguistic functions to differentiate between the risk profile for dyslexia and learning difficulties. Statistically significant changes were observed in measures of phonological awareness, lexical access speed, accuracy in decoding and encoding (hit rate, rate, and typology of errors in reading and writing), reading speed, and textual comprehension. The gains obtained in phonological awareness, increase in text reading speed, and reading and writing accuracy, expressed in a decrease in the error rate and error typology, were significant for differentiating performance profiles.

Working memory was the only function in which no changes were observed in the post-intervention assessment. It is already well known that difficulties in consolidating reading are associated with a short span of digits^(22)^, with the phonological loop of Baddeley's model^(23)^ being responsible for allowing verbal items to be mentally maintained during the manipulation necessary for performance and emission of responses, as observed in rhyme judgment and pseudoword repetition tasks. In Maehler's study^(24)^, no changes in performance on verbal working memory tasks were observed after an intervention program focusing on reading. The author attributed these results to a low sensitivity of training on memory skills, reflecting that more ecological strategies, which consider the child's daily functioning, could be more effective.

Our findings revealed higher scores on the rapid automatic naming task-RAN after the intervention, which indicates an increase in lexical access speed in the sample. However, participants with a risk profile for dyslexia did not differ significantly from the others, which suggests that this ability did not discriminate between the groups. However, previous evidence indicates that dyslexic children are commonly slower in their lexical access speed^(25)^. A possible explanation for our findings is that rapid automatic naming tasks require the integration of several cognitive resources, such as attention and processing speed. Thus, performance variability may have been influenced by cognitive weaknesses other than naming ability.

The changes in measures of phonological awareness and decoding/coding skills proved to be especially important in identifying children at risk for dyslexia. For example, a gain of 33% in performance of phonological awareness task was observed among participants considered to be at no risk. A development of this ability in children with reading and writing difficulties undergoing phonological and multisensory stimulation programs has widely been reported^(26,27)^

In the reading task, our findings revealed an improvement in decoding accuracy regardless of the level of frequency (high and low) and lexicality (words and pseudowords), in addition to a reduction in the error rate in the post-intervention stage of the sample. In monitoring tasks, there were gains only in reading pseudowords. Tilanus et al.^(28)^ also observed better reading accuracy of pseudowords compared to regular words in children with a risk profile for dyslexia after a 12-week intervention with an emphasis on grapheme-phoneme correspondence training. In our study, despite the constant progress observed in pseudowords, we still found greater difficulties in decoding these stimuli compared to high-frequency words. Differences in the reading accuracy of words and pseudowords are expected given that high-frequency words are usually read more easily. The more frequent the exposure to a word, the greater the possibility of storage in the semantic and orthographic lexicon, leading primarily to reading via the lexical route. This process does not occur with pseudowords, as they require access to the phonological lexicon^(6,8)^. When comparing the groups, we found that non-dyslexics showed a 17.44% greater reduction in post-intervention errors, compared to the others. Participants with a risk profile for dyslexia did not show substantial changes in the number of errors made. Furthermore, an important difference was observed between accuracy and reading speed in monitoring assessments. Although a substantial increase in the number of words read correctly was not observed, participants showed greater reading speed, although at levels still below those expected for their age group and education. This finding can be justified by the degree of difficulties identified at the beginning of the intervention and also by the deficit in phonological skills^(5)^ that make decoding difficult. Considering that the lexical route is essential for reading fluency, as it favors textual comprehension^(5,6)^, it was expected that with low fluency performance, text comprehension would be equally difficult. Both speed and comprehension demand well-developed cognitive-linguistic processes, including syntactic, semantic, inferential components, and memory integration.

The improvement in writing words was evident more in a reduction in errors than in an increase in correct answers. In other words, participants started to make fewer mistakes in phoneme-grapheme correspondences, a more significant improvement than the increase in correct answers in the global word. The frequency of errors after the intervention was 16.48% lower among participants without a profile at risk for dyslexia. Elective intervention effects in phonics instruction in terms of reduction in the rate of decoding errors in both words and pseudowords have also been reported in international studies^(28)^. Brazilian studies^(29,30)^ involving elementary school children in general reported only an increase in correct answers. In our study, we found that exploring types of errors can offer indicators of the strategy used by the child in reading and writing. For example, irregular words can only be accessed through the lexical route, since the correspondence is arbitrary and the child must memorize how the word is pronounced^(6)^. When the stock of words stored in the orthographic lexicon is reduced because of less exposure to reading, language regularization and/or contextual errors may occur. These errors were observed more frequently among children with learning difficulties after the intervention. Participants with a risk profile for dyslexia made more errors due to the substitution of deaf-sound phonemic pairs, both in reading and writing, which may indicate losses in the phonological route, important for accuracy in graphophonemic conversion. These findings were similar to other studies that compared the types of errors in reading and writing between dyslexic and non-dyslexic samples^(29,30)^.

According to the traditional RTI model^(2,3)^, as soon as a child at risk is identified, their ability to respond to intervention must be monitored. Our study used monitoring as a process of systematic and continuous assessment of performance in reading and writing, to understand the learning curve. Differences in the profile of this curve were one of the parameters to discriminate children with a dyslexia risk profile from those with learning difficulties. The application of standardized instruments to investigate cognitive-linguistic skills before and after the intervention also generated measures of performance gains. Another important aspect is that a single variable to measure the child's responsiveness proved to be indiscriminate, probably because learning is a process that encompasses different skills. Therefore, qualitative-quantitative analyses have greater potential to deal with the heterogeneity of learning difficulties.

The evolution in phonological skills was particularly important for understanding reading and writing difficulties. Participants with a risk profile for dyslexia, although they improved, demonstrated greater effort throughout the sessions and needed more time to automate graphophonemic correspondence strategies, being considered low responders. Brazilian^(3)^ and international^(26)^ studies based on the traditional RTI model also address phonological and decoding skills as important measures to discriminate between responding and low-responding children. The observations of the present study also suggest that the risk profile for dyslexia is associated with low responsiveness, as evidenced by an established (minimum) rate of variation of less than 25%, regarding linguistic skills and decoding errors. The profile also appears to be associated with oscillating learning curves, identified through performance monitoring. That is, the rate of gain achieved in the intervention, that is, how much performance changed after the intervention in comparison with the initial result, was lower among children identified with a risk profile for dyslexia. It should be emphasized that these data do not indicate that they did not improve, but that they showed slower evolution. Longitudinal studies also showed that dyslexic children tend to maintain a slow and non-automatic reading and writing pattern^(5,31)^. The DSM-5^(1)^ also describes this evolution profile in children with dyslexia categorizing low response accuracy as one of the diagnostic criteria. This occurs because the difficulty is persistent and achieving performance, even if average, requires great effort and constant mediation. This pattern sets up a discrepancy between what is expected in terms of response to interventions in reading and writing and what the child has managed to evolve, approaching the RTI assumptions.

Our findings reinforce previous reports that the cognitive expressions of dyslexia are heterogeneous but learning difficulties can also vary substantially in terms of manifestations and causes. Participants without critical signs of dyslexia showed some performance characteristics similar (but not specific) to those of the others but with different etiologies. We identified that in 60% of cases, the difficulties were justified by behavioral and psychopedagogical issues, the need for adaptations to school contexts, and low motivation or associated with other neurodevelopmental disorders. We also identified that 77.8% of children with learning difficulties came from public schools. These results may reflect socioeconomic and environmental differences, including the weaknesses of the Brazilian educational system. Therefore, the need for early identification and intervention in children who present learning difficulties in elementary school is reinforced, aiming to minimize the emotional and behavioral repercussions.

From this perspective, our findings indicate that the response to intervention approach constitutes a more valid alternative for identifying children at risk for dyslexia than one based on a single assessment of school performance and cognitive functions, avoiding false-positive cases and/or waiting for diagnosis. Our findings reinforce that a model of response to intervention associated with diagnostic processes must follow steps such as screening children to identify risk symptoms for dyslexia, intervention in cognitive-linguistic skills, performance monitoring, and reassessment. To discriminate children with dyslexia through the analysis of responsiveness to intervention, the following procedures proved to be facilitating^(1)^: analyze changes in phonological awareness measures and quantify them considering gain parameters^(2)^; analyze the pattern of the learning curve in reading and writing measures, primarily the error rate^(3)^; identify a typology of errors, mainly the persistence of substitution of deaf-sound phonemic pairs. The scheme of 12 weekly sessions lasting two hours and the group configuration of five participants proved to be viable for detecting cases at risk for dyslexia. The characteristic of sessions with an evaluative and remedial nature aligned with monitoring changes in performance seems to allow greater diagnostic security. Additionally, the importance of a multidisciplinary approach in working with children with low academic performance around health and education is reinforced.

As limitations of the study, we consider that the parameters (cutoff point) used to quantify and qualify responsiveness to intervention may not be useful in all contexts and for all children, due to the heterogeneity of learning difficulties. In other words, although cut-off points in quantitative measures can help to discriminate between good and bad readers, they are still arbitrary. It is known that academic skills are distributed along a continuum that encompasses the ability and inability to read, a concept called the dimensional model^(1)^. We understand that the diagnostic-interventional program proposed in this study is closer to a dimensional rather than a categorical diagnostic perspective based on the idea of discontinuity, that is, a rupture between a good reader and a “poor reader”^(1)^. Future research may propose other discrimination criteria, given the need to improve diagnostic processes.

## CONCLUSION

This study developed and analyzed the suitability of an intervention program for reading and writing difficulties based on the Response to Intervention model. The program involved the stimulation of phonological skills and systematic teaching of graphophonemic correspondences, monitoring of acquisitions, and pre- and post-intervention assessment. Phonological awareness skills, reading speed and frequency, and types of errors in reading and writing were those that proved to be most appropriate in identifying the 12 children with a risk profile for dyslexia. Programs of this nature can encourage dialogue between health and education professionals to care for elementary school students as well as avoid the risk of false positives in dyslexia in the clinical context. More research is needed to analyze intervention response models aimed at an assertive diagnosis of dyslexia and, especially, on the applicability of the RTI model in Brazil.
