# Supplementary material for: Response to intervention as an identification strategy of the risk for dyslexia
Source: Codas. 2024 Jun 10;36(4):e20230031. doi: 10.1590/2317-1782/20242023031en (PMC11189365; doi:10.1590/2317-1782/20242023031en)
Supplement: Translation

# Translation: Resposta à intervenção como estratégia de identificação do risco para dislexia

**DOI:** 10.1590/2317-1782/20242023031pt

**Authors:** Mariana Gobbo Medda, Thais Barbosa, Isadora Salvador Rocco, Claudia Berlim de Mello

**Affiliations:** 1 Departamento de Psicobiologia, Universidade Federal de São Paulo – UNIFESP - São Paulo (SP), Brasil.

**Keywords:** Dislexia, Transtorno Específicos de Aprendizagem, Intervenção, Aprendizagem, Problemas de Aprendizagem

## Abstract

**Objetivo:**

desenvolver um processo de intervenção para identificação de crianças em risco para dislexia, baseado no modelo de resposta à intervenção. Especificamente, identificar o perfil de mudança no desempenho pós-intervenção em tarefas de consciência fonológica, memória operacional, acesso lexical, leitura, escrita; e analisar quais funções cognitivas tiveram efeito significativo para discriminação de estudantes em risco para dislexia.

**Método:**

amostra composta por 30 participantes com dificuldades na leitura e escrita, entre 8 e 11 anos, de escola pública ou particular, estudantes do 3º ao 5º ano. Todos foram submetidos a uma bateria de testes cognitivo-linguísticos, antes e após a realização de 12 sessões de intervenção. Para monitoramento do desempenho foram aplicadas cinco listas de leitura e escrita de palavras/pseudopalavras. Foram realizadas análises, qualitativas e quantitativas, das diferenças de desempenho pré e pós-intervenção; e entre os participantes na pós avaliação, para compreensão de grupos perfil em risco para dislexia vs não-dislexia.

**Resultados:**

ocorreram mudanças estatisticamente significativas em nomeação automática rápida, compreensão de texto, consciência fonológica, taxa e tipologia de acertos/erros na leitura/escrita e velocidade de leitura. Sendo essas três últimas variáveis as que se mostraram mais discriminativas dos grupos, todas com menos ganhos na pós-intervenção para o grupo com perfil em risco de dislexia.

**Conclusão:**

a intervenção com foco na estimulação das habilidades fonológicas e ensino explícito das correspondências grafofonêmicas contribuiu para a evolução dos participantes. A abordagem de resposta à intervenção favoreceu a identificação de crianças com perfil em risco para dislexia, as diferenciando de crianças com outras dificuldades de aprendizagem.

## INTRODUÇÃO

O transtorno específico da aprendizagem com prejuízo na leitura - Dislexia - é o tipo mais comum de transtorno de aprendizagem. O Manual Diagnóstico e Estatístico de Transtornos Mentais - DSM 5^(1)^ define dislexia como uma condição de origem neurobiológica, caracterizada por um padrão de dificuldades persistentes na decodificação, ortografia e fluência de leitura. Difere assim das dificuldades de aprendizagem (DA) na leitura e na escrita que decorrem de fatores extrínsecos, tais como condições socioeconômicas desfavoráveis, ineficiência educacional, falta de estimulação adequada no ambiente familiar, fatores de ordem emocional-afetiva ou secundários à presença de outros diagnósticos.

Em vista da heterogeneidade das causas das DA e reconhecendo as limitações diagnósticas, estudos^(2,3)^ destacam a relevância de modelos de prevenção associados à identificação precoce de sinais de risco para dislexia. Esses modelos são denominados de Resposta à Intervenção (RTI), que em geral pressupõem um conjunto de processos avaliativos e remediativos. Apesar das variações, o RTI tradicional é aplicado na escola, sendo o modelo de três camadas o mais difundido. Por camadas entende-se as diferentes fases de implementação, caracterizadas por focos específicos e cada vez mais intensivos de intervenção. O estudante é submetido à avaliação das aquisições atuais em leitura e escrita, intervenção e reavaliação com desempenho monitorado continuamente para observar mudanças na taxa de aprendizado. Ao final de cada camada, a ausência de melhora no desempenho ou evolução lenta e insuficiente poderá indicar baixa resposta à intervenção. A persistência desse padrão é um dos requisitos diagnósticos da dislexia^(3)^_._

Os modelos de intervenção com base no processamento fonológico têm sido os mais recomendados para crianças com desempenho de risco para dislexia visto que as dificuldades de consciência fonológica, memória operacional verbal e velocidade de acesso lexical são apontadas como a tríade sintomática^(4)^. Há evidências de que a estimulação sistemática e explícita das habilidades fonológicas combinada à instrução da correspondência grafema-fonema auxilia a compreensão do princípio alfabético, bem como o desenvolvimento adequado das habilidades metafonológicas, favorecendo a aquisição da leitura^(5-7)^. Um dos métodos de ensino da leitura e escrita é o fônico, que estimula a consciência fonológica e a estimulação multissensorial^(8)^. Esta última combina diferentes modalidades sensoriais para estimular a criança a estabelecer conexões entre aspectos visuais (ortografia da palavra), auditivos (fonologia), táteis (memória tátil da forma das letras/palavras), sinestésicos da grafia (percepção das coordenadas necessárias para escrever) e da articulação (movimentos para pronunciar sons de forma consciente e intencional).

Em países em desenvolvimento como o Brasil, entretanto, a implementação dos programas de RTI tem recebido pouca ênfase, apesar das iniciativas de pesquisadores^(3,9)^. Nas escolas públicas, em especial, o elevado número de alunos por sala traz para os professores muitos desafios para adotar estratégias pedagógicas individualizadas. Assim, o procedimento recorrente para lidar com o alarmante número de escolares com baixo rendimento acadêmico tem sido o encaminhamento a serviços diagnósticos especializados que realizam avaliações pontuais, que geralmente envolvem testes formais de habilidades cognitivas e de aquisições acadêmicas com base na idade cronológica e grau de escolaridade. Uma consequência de avaliações desta natureza pode ser a ocorrência de disléxicos falso-positivos.

Em suma, precocidade de intervenções baseadas em estimulação cognitivo-linguística, monitoramento do desempenho e compartilhamento dos desafios pedagógicos entre profissionais são características essenciais do RTI. Um dos benefícios potenciais é evitar o alto número de encaminhamentos para avaliação diagnóstica por profissionais de áreas da saúde, em contexto clínico, anterior a uma abordagem pedagógica em ambiente escolar. Embora indicados em situações específicas, tais processos avaliativos deveriam considerar como os indivíduos respondem à intervenção prévia, incorporando assim estratégias de avaliação e intervenção multidisciplinar^(2)^_._ Trata-se de uma perspectiva consonante com as diretrizes do DSM-5^(1)^ para diagnóstico da dislexia, que pressupõem persistência de sinais de risco ou dificuldades específicas após intervenção sistemática.

Entende-se que modelos desta natureza também podem contribuir para um refinamento de processos diagnósticos em contexto clínico. Ou seja, uma abordagem integrando ações de avaliação e intervenção pode favorecer a discriminação entre dificuldades de aprendizagem e um perfil sugestivo de transtorno especifico como a dislexia, o que é fundamental para a adequação pedagógica.

Nessa perspectiva, o objetivo deste estudo foi desenvolver um processo de intervenção para identificação de crianças em risco para dislexia baseado no modelo de resposta à intervenção para estudantes no Ensino Fundamental I. O processo priorizou um dos pressupostos básicos do RTI, que é estimular habilidades cognitivo-linguísticas em crianças com dificuldades escolares e verificar como respondem às estratégias, antes do diagnóstico. O programa foi estruturado em etapas e envolveu processos de avaliação e de intervenção com foco em habilidades cognitivo-linguísticas, à semelhança da camada 2 do RTI original. Ao observar como as crianças respondem às estratégias ministradas, é possível assim melhor direcionar o encaminhamento de intervenções seja em âmbito terapêutico seja pedagógico. Ou seja, embora baseada em parâmetros fundamentais, a proposta não implica em uma relação direta com o modelo RTI original, aplicado em escolas. Por outro lado, o programa se organiza em uma abordagem multidisciplinar na medida em que demanda uma articulação de ações entre profissionais de educação e saúde, como fonoaudiólogos e psicólogos.

## MÉTODO

O delineamento do estudo foi observacional analítico, transversal, com medidas repetidas. Foram respeitadas as diretrizes regulamentadoras para pesquisa com seres humanos conforme a Resolução 466/12 do Conselho Nacional de Saúde. O projeto foi previamente aprovado pelo Comitê de Ética em Pesquisa (parecer n° 134234/2018). As crianças e seus responsáveis foram informados quanto aos propósitos e procedimentos do estudo e Termos de Consentimento Livre e Esclarecido (TCLE) e de Assentimento foram assinados antes da coleta dos dados.

### Participantes

Participaram do estudo 30 crianças com perfil de desempenho em leitura e escrita identificado como de risco para dislexia, entre 8 e 11 anos de idade, do 3º ao 5º ano do Ensino Fundamental I de escolas das redes pública e particular de São Paulo. Todas foram recrutadas dentre as encaminhadas para avaliação no Núcleo de Atendimento Neuropsicológico Infantil (NANI), em função de queixas de dificuldades escolares. O NANI é uma das unidades do Centro Paulista de Neuropsicologia, instituto apoiado pela Associação Fundo de Incentivo à Pesquisa (AFIP) e vinculado academicamente ao Departamento de Psicobiologia da Universidade Federal de São Paulo/UNIFESP, desenvolve ações em ensino e pesquisa com foco na abordagem multidisciplinar de transtornos do neurodesenvolvimento. A busca por avaliação diagnóstica no NANI em geral se dá por encaminhamento de serviços médicos ou dos educadores, incluindo as crianças cujas famílias buscaram espontaneamente por avaliação diagnóstica ou encaminhadas pela escola, considerando como queixas principais dificuldades de aprendizagem.

Os indicadores de risco para dislexia foram baseados em dados do histórico prévio: (a) presença de dificuldades desde o início da escolarização conforme documentado em relatórios escolares e relato dos responsáveis e (b) ter participado de atividades diferenciadas com foco na leitura e escrita no ambiente escolar; e em dados da avaliação de habilidades cognitivo-linguísticas: (c) desempenho inferior em testes padronizados de consciência fonológica, de memória operacional verbal, de velocidade de acesso lexical, de leitura e escrita de palavras, pseudopalavras e de texto e (d) evidências de padrão atípico de erros nos testes de leitura e escrita.

Os critérios de exclusão consideraram histórico de intercorrências pré, peri ou pós-natais de risco para alterações neurológicas, como nascimento anterior à 36ª semana de gestação; diagnóstico de autismo e/ou de dislexia já estabelecidos (informados pela família), de deficiência intelectual (QI < 70) ou doenças neurológicas, genéticas ou psiquiátricas; presença de alterações de acuidade visual ou auditiva não corrigidas. Crianças com sinais de risco para transtorno de déficit de atenção-hiperatividade ou de linguagem não foram excluídas da amostra dada a alta prevalência de comorbidade com dislexia. Nenhuma criança selecionada para o estudo fazia uso regular de medicações com ação no sistema nervoso central. A identificação de sinais de risco para os diferentes quadros do neurodesenvolvimento ocorreu após a avaliação pré-intervenção (processo inicial do estudo).

### Procedimentos

Um total de 68 crianças foram consideradas inicialmente elegíveis para o estudo com base nos indicadores de histórico prévio e submetidas à avaliação pré-intervenção. Dentre estas, 38 não preencheram critérios de inclusão baseados no desempenho nos testes cognitivo-linguísticos e não foram incluídas no estudo, mas continuaram a avaliação multiprofissional no NANI. Portanto, uma amostra final de 30 crianças foi submetida ao processo interventivo.

### Caracterização do programa de avaliação-intervenção

Diferentemente do modelo tradicional (ou seja, realizado em contexto escolar e em três níveis de intervenção), o programa foi delineado tendo como base os parâmetros da Camada 2 do RTI. Foram estipuladas três etapas em perspectiva tanto avaliativa como remediativa, a citar^(1)^: avaliação pré intervenção^(2)^; intervenção e monitoramento do desempenho; e^(3)^ avaliação pós intervenção (ver Figura 1). Todas as etapas foram conduzidas por especialistas em neuropsicologia, fonoaudiologia e/ou psicopedagogia.

**Figura 1:** Esquema geral das etapas do estudo

### Avaliação pré e pós intervenção

Em entrevista inicial, os responsáveis forneceram informações do histórico do neurodesenvolvimento, clínico e educacional, e responderam à versão brasileira do Child Behavior Checklist *-* CBCL^(10)^. Na sequência, as crianças foram submetidas à Escala Wechsler Abreviada de Inteligência-WASI^(11)^._._ Estes procedimentos visaram especificamente o atendimento aos critérios de exclusão adotados. A avaliação pré e pós intervenção incluiu a investigação de habilidades cognitivo-linguísticas por meio de instrumentos desenvolvidos para esta finalidade, como segue. Foram usados para avaliar a memória operacional os Testes de Repetição de Palavras e Pseudopalavras^(12)^, tarefas *span* de dígitos e *span* de Blocos-Corsi (repetição em ordem inversa)^(13)^. Velocidade de acesso lexical foi analisada por meio do Teste de Nomeação Automática Rápida de objetos, cores, números, letras, ou seja, *Rapid Assessment Naming*-RAN^(14)^. Habilidades de consciência silábica e fonêmica foram avaliadas por meio do teste Consciência fonológica por produção oral^(15)^. Na avaliação das competências em leitura e escrita foram usadas as Provas de Avaliação dos Processos de leitura-PROLEC^(16)^, subtestes de Leitura de Palavras e Pseudopalavras, considerando a taxa de acertos para palavras (baixa e alta frequência) e pseudopalavras, bem como a taxa de erros (total de fonemas de cada estímulo). A velocidade e compreensão da leitura em voz alta foram avaliadas com base em dois textos do Subteste Compreensão de Textos-PROLEC^(16)^. A avaliação teve como foco o processo semântico (porcentagem de questões respondidas corretamente e o número de palavras lidas por minuto). Para a avaliação da escrita foram analisadas taxa de acertos e de erros e tipologia de erros do teste Escrita sob Ditado^(17)^. Os erros na leitura e escrita foram categorizados conforme tipologia específica^(18)^: lexicalização, substituição de fonemas surdo-sonoros, inversões, omissões e/ou acréscimos, desconhecimento de regra contextual e ortografia, troca de vogais, outros erros. Por fim, para avaliar a estrutura narrativa a criança foi solicitada a escrever uma história a partir de uma sequência de imagens. A análise seguiu critérios categóricos sugeridos nos estudos de Spinillo e colaboradores^(19)^, com adaptação para este estudo: não resposta (recusa em escrever), não história (escrita de frases pouco conectadas), produção descritiva (sem construção de sequência de acontecimentos ou relações causais), história completa (estrutura narrativa elaborada, podendo apresentar vocabulário mais restrito e produção curta, desde que apresente início, meio e fim, apresentação do conflito e desfecho).

### Intervenção

Os 30 participantes foram alocados em grupos de cinco crianças, visto que as intervenções e observações de respostas seriam mais eficazes em grupos menores. O agrupamento levou em consideração a idade cronológica e o horário escolar de cada participante, uma vez que as intervenções foram no contraturno. Outros critérios foram considerados, quando possível, como o nível de desempenho na leitura e escrita, que na amostra variou entre aquisições mais básicas, como reconhecimento de palavras isoladas, até uma capacidade elementar de compreensão textual. Importante mencionar que um agrupamento estrito conforme nível de leitura mostrou-se inviável dada a heterogeneidade do desempenho da amostra, mas que reflete a realidade nas salas de aula.

A intervenção foi delineada como uma abordagem estruturada e sistemática de estimulação de habilidades de codificação e decodificação de leitura e escrita. A estimulação foi multissensorial, com o objetivo inicial de fornecer estratégias de base para discriminação entre os fonemas, ensinando suas características auditivas e articulatórias, necessárias para que, posteriormente, as crianças fossem capazes de representar sequências de sons em sílabas. As tarefas seguiram uma ordem de complexidade, de conceitos mais básicos para os mais complexos. Materiais concretos, como papel-cartão, letras móveis e espelho foram utilizados como facilitadores. Atividades orais foram sempre alinhadas a associações com figuras de diferentes categorias. Os participantes foram frequentemente estimulados a refletir sobre as próprias respostas e desempenho, tornando-se capazes de se autocorrigir.

A intervenção foi estruturada em 12 sessões de duas horas, uma a cada semana. A periodicidade das sessões deveu-se às possibilidades das famílias para comparecer ao serviço. Tanto a organização das sessões como as tarefas ministradas foram baseadas e adaptadas de estudos com aplicabilidade do RTI na escola, como em Andrade e Capellini^(3)^, Almeida e colaboradores^(9)^; e em outros programas de intervenção como os de Seabra, Capovilla^(8)^.

Nas primeiras seis sessões o foco foi o ensino explícito e sistemático de habilidades mais básicas de correspondência fonema-grafema por meio da estimulação multissensorial, com o uso de estratégias fonovisuoarticulatórias e de estimulação fônica. Nesta etapa foram trabalhados aspectos da linguagem oral, como consciência fonológica, discriminação de fonemas (com foco nos pares surdo-sonoros, fornecendo dicas articulatórias), memória auditiva/verbal, como repetição e manipulação de informações verbais, categorização, nomeação e vocabulário. A proposta foi explorar as características semânticas das palavras e seu uso em frases simples, seguindo para complexas e o uso do vocabulário específico em narrativas orais estruturadas. Nas seis sessões seguintes, foi dada ênfase a elementos mais complexos, tendo como foco principal a escrita e a leitura de frases e textos. A leitura foi estimulada principalmente em voz alta e compartilhada, com menor demanda de leitura silenciosa e individual. Utilizou-se de estratégias de estimulação da rota fonológica, com ensino explícito das correspondências, e lexical, por exemplo, com listas de palavras que compartilham de regras ortográficas e suas exceções, estimulando também a memória visual das palavras. Procurou-se intercalar as tarefas com ênfase na estimulação de habilidades cognitivo-linguísticas, a exemplo de leitura de palavras ou memória operacional, com atividades mais lúdicas, curtas e compartilhadas, como jogos, ou pequenos intervalos, a fim de minimizar cansaço ou perda de engajamento.

### Monitoramento do desempenho ao longo da intervenção

Para monitorar o desempenho dos participantes foram coletados dados de avaliações de leitura e escrita de cinco listas de palavras e pseudopalavras, aplicadas quinzenalmente, em sessão individual. As listas foram compostas por 15 itens selecionados do estudo de Pinheiro^(20)^, sendo cinco palavras de alta frequência, cinco de baixa frequência e cinco pseudopalavras. Os estímulos variaram a cada testagem, mantendo características psicolinguísticas quanto à regularização, lexicalização e frequência. O monitoramento visou uma análise da curva de aprendizagem de cada criança, considerando acurácia (taxa de acertos, de erros e tipologia de erros na leitura e na escrita) e velocidade de leitura.

### Análise dos dados

A análise dos dados foi conduzida em três etapas. A primeira envolveu abordagem de modelos mistos de medidas repetidas (Generalized Mixed Model - GMM) foi conduzida usando o programa IBM SPSS 21. Para cada variável foi testado o efeito do tempo (análise de comparação entre A1 - pré-intervenção e A2 - pós intervenção) e análise dos cinco tempos (cinco ocorrências de avaliação do monitoramento do desempenho, L1 a L5), com vistas a identificação de variáveis com mudança significativa, considerando toda amostra (n=30). Sendo assim, tivemos ao todo sete momentos de avaliação (pré e pós intervenção e cinco avaliações de monitoramento). A segunda etapa teve foco na definição de responsividade à intervenção. Foram calculadas as taxas de variação individual (taxa delta) a fim de identificar o quanto cada indivíduo mudou na A2 de acordo com o seu próprio desempenho na fase pré intervenção (foram analisadas nessa etapa apenas as variáveis em nível significante de p<.05 na comparação pré e pós de toda a amostra). Esses valores foram organizados em mediana e intervalos interquartil. Assim, foi considerado que os participantes que não conseguiram ultrapassar o limite inferior (ou seja, quartil 25%) na taxa de variação, apresentaram baixa resposta à intervenção. Consideramos o percentil 25 como indicador de baixa responsividade, conforme indicadores clínicos baseados em propriedades psicométricas^(21)^. Considerou-se também a análise da curva de percentagem de erros e acertos na leitura e na escrita de palavras e pseudopalavras na etapa de monitoramento, bem como a tipologia de erros, e análises qualitativas do desempenho. Com base nas análises de resposta à intervenção desta etapa, os 30 participantes foram classificados em dois grupos: participantes com perfil de risco para dislexia (GD n=12) *versus* não-dislexia (GND n=18). Para análise de responsividade dos participantes e discriminação dos grupos, considerou-se as variáveis (a) consciência fonológica, (b) acesso lexical, (c) taxa de erros e acertos em leitura/escrita, (d) tempo e acurácia de leitura, (e) tipologia de erros na leitura e escrita. Por fim, na terceira etapa um modelo generalizado (Teste estatístico Generalized Linear Model - GLzM), controlado pela idade (covariável), foi conduzido com o objetivo de verificar possíveis diferenças entre os grupos considerados em situação pós intervenção. Os pré-requisitos foram testados para todas as variáveis dependentes e a significância se deu no nível geral de p<,05.

## RESULTADOS

Segundo caracterização sociodemográfica da amostra, a média de idade foi de 9,2 anos (DP=,94), com predomínio de meninos (60%) e de alunos de escola pública (66,7%), sendo 36,7% estudantes do 3º ano, 33,3% do 4º e 30% do 5º ano.

### Comparação das medidas pré e pós-intervenção

As Tabelas 1 e 2 apresentam os dados referentes à Etapa 1 de análise de dados, e expressam diferenças de desempenho nos domínios de memória operacional, acesso lexical, consciência fonológica, leitura e escrita entre as avaliações pré e pós intervenção, na amostra como um todo (n=30). Diferenças estatisticamente significantes foram observadas nos testes de acesso lexical, ou seja, nomeação de objetos (F=5,66/p=,024), números (F=5,21/p=,030) e letras (F=10,02/p=,004); de consciência fonológica (F=35,16; p<,001), nos domínios silábico, em manipulação silábica (F=8,76/p=,005) e aliteração (F=4,60/p=,040); e fonêmico em síntese (F=9,05/p=,005); segmentação (F=19,33/p<,001) e manipulação (F=4,79/p=,037), com melhora na A2, conforme pode ser observado na Tabela 1.

**Tabela 1:** Diferença do desempenho da amostra total na avaliação pré e pós intervenção para os domínios: Memória Operacional; Acesso Lexical; Consciência Fonológica

Domínio	Variável^a^		Média (Desvio-Padrão)		Intervalo de confiança 95%		Estimativas^b^	t	Sig.
			**Pré**	**Pós**	**Pré**	**Pós**			
Memória operacional	Dígitos		49,10(2,0)	50,00(1,6)	45,0-53,1	46,5-53,4	0,90	-,365	,718
	Corsi		48,80(1,8)	52,83(1,8)	45,0-52,5	49,1-56,5	4,03	-1,96	,059
	TRP		49,05(2,5)	53,00(2,6)	43,6-54,4	47,5-58,4	3,95	-2,05	,054
	TRPP		50,81(1,4)	49,05(1,8)	47,8-53,7	45,1-52,9	-1,76	1,04	,310
Acesso Lexical	RAN objetos		39,80(1,4)	42,80(1,4)	36,9-42,6	39,9-45,6	3,00	2,38	,024*
	RAN cores		39,53(1,4)	39,13(1,5)	36,5-42,5	36,0-42,2	-,40	-,34	,734
	RAN números		38,53(1,1)	40,66(1,1)	36,1-40,9	38,2-43,0	2,13	2,28	,030*
	RAN letras		36,10(1,4)	39,70(1,4)	33,2-38,9	36,8-42,5	3,60	3,16	,004*
Consciência Fonológica	Silábica	Síntese	47,07(2,2)	50,20(1,3)	42,4-51,6	47,5-52,8	3,13	1,39	,175
		Segmentação	52,10(,98)	52,93(,98)	50,1-54,0	50,9-54,9	-,83	-,71	,478
		Manipulação	49,23(1,7)	55,20(1,0)	45,7-52,7	53,0-57,3	5,96	2,96	,005*
		Transposição	49,13(1,5)	51,50(1,4)	46,0-52,2	48,5-54,5	2,36	1,32	,197
		Rima	40,47(2,5)	43,73(2,4)	35,2-45,6	38,7-48,7	3,26	1,40	,172
		Aliteração	40,50(2,6)	47,13(2,6)	35,1–45,8	41,7-52,4	6,63	2,14	,040*
	Fonêmica	Síntese	38,86(1,4)	45,70(2,0)	35,9-41,8	41,5- 49,8	6,83	3,00	,005*
		Segmentação	43,30(1,5)	51,00(1,7)	40,1–46,4	47,5-54,4	7,70	4,39	<,001*
		Manipulação	40,06(2,0)	44,50(2,0)	35,9-44,1	40,3-48,6	4,43	2,18	,037*
		Transposição	42,80(1,3)	45,76(2,0)	40,1-45,4	41,6-49,8	2,96	1,65	,109
	C.F. Total		37,13(2,2)	46,60(2,30)	32,4-41,8	41,8-51,3	9,46	5,93	<,001*

Teste estatístico Generalized Mixed Model (GMM)

* Diferença média é significativa no nível ,05

a Variável dependente: Dígitos e Corsi - *Span* ordem inversa;

b Diferença Média

**Legenda:** TRP=Teste de Repetição de Palavras – acertos totais; TRPP=Teste de Repetição de Pseudopalavras – acertos totais; RAN=nomeação automática rápida – tempo total de nomeação em segundos; C.F. Total=Consciência fonológica acertos totais

Houve diferenças entre os índices de leitura e escrita de palavras e pseudopalavras obtidas entre A1 e A2 (Tabela 2). Na leitura ocorreu aumento de 16,94 pontos em média na taxa de acertos totais (F=36,31/p<,001); nas taxas de acertos em palavras de alta (F=12,14/p=,002), baixa frequência (F=8,34/p=,007) e pseudopalavras (F=7,16/p=,012). A taxa de erros diminuiu 14,37 pontos em média (F=10,27/p=,003). Ocorreu diferença no número de palavras lidas por minuto (PPM) em texto narrativo (F=12,17/p=,003) e texto expositivo (F=14,54/p=,001). Estes avanços parecem ter favorecido a compreensão leitora do texto narrativo (F=14,49/p=,001), visto o aumento no número de acertos nos questionários. Na escrita ocorreu aumento de 11,36 pontos em média na taxa de acertos (F=35,09/p<,001)*,* com ganho de 11,26 pontos em média em palavras (F=24,59/p=<,001) e de 12,63 pontos em média em pseudopalavras (F=18,23/p=<,001). A taxa de erros totais diminuiu significativamente (F=13,14/p=,001), em palavras (F=6,57/p=,016) e em pseudopalavras (F=8,85/p<,001), os participantes tiveram ganhos na pontuação padrão dos testes. Em tarefa de redação, houve progresso com transição de uma categoria a outra (F=11,9/p=,002), por exemplo, na A1, 33,3% da amostra desempenhou na categoria inicial /não-respostas/, já na A2, o predomínio foi de 60% de /produções completas/, representando um incremento de 50% na frequência total para essa categoria. Na A1, 30% da amostra produziu histórias /descritivas/ e na A2, 6,7% se mantiveram nessa categoria.

**Tabela 2:** Diferença do desempenho da amostra total na avaliação pré e pós intervenção para os domínios de Leitura e de Escrita

**Domínio**		**Variável** a	**Média (Desvio Padrão)**		**Intervalo de confiança 95%**		**Estimativas** b	**t**	**Sig.**
			**Pré**	**Pós**	**Pré**	**Pós**			
Leitura	Acertos	Total	52,29(4,7)	69,23 (4,7)	42,5-62,0	59,5-78,9	16,94	6,02	,001*
		Pal.AF	30,20(2,6)	38,26 (2,6)	24,8–35,5	32,9–43,6	8,06	3,48	,002*
		Pal.BF	23,03(1,2)	29,33(2,3)	20,4-25,6	24,5-34,1	6,30	2,88	,007*
		PP	21,53(1,3)	25,16(1,3)	18,8-24,1	22,5-27,8	3,63	2,67	,012*
	Erros	Total	24,34(4,5)	9,97(4,5)	15,1-33,5	,74 -19,2	-14,37	-3,20	,003*
	Texto Nar	PPM	46,89(5,2)	57,67(5,0)	36,1-57,6	47,3-68,0	10,77	3,48	,003*
		CO	48,29(7,5)	71,87(7,1)	32,9-63,6	57,1-86,5	23,58	3,77	,001*
	Texto Exp	PPM	36,22(4,7)	45,18(4,6)	26,5-45,8	35,6-54,6	8,96	3,81	,001*
		CO	72,65(5,4)	69,79(6,2)	60,4-84,8	56,9-82,6	-2,86	-,587	,569
Escrita	Acertos	Total	29,73(4,5)	41,10(4,5)	20,4-38,9	31,8-50,3	11,36	5,92	<,001*
		Pal.	30,10(4,7)	41,36(4,3)	20,2-39,9	32,4-50,3	11,26	4,96	<,001*
		PP.	28,16(4,8)	40,80(4,8)	18,3-37,9	30,9-50,6	12,63	4,27	<,001*
	Erros	Total	35,53(5,0)	21,66(5,0)	25,2-45,7	11,4-31,9	-13,86	-3,62	,001*
		Pal.	27,76(2,1)	30,06(2,1)	23,3-32,2	25,6-34,5	2,30	2,56	,016
		PP.	30,06(2,6)	34,46(2,6)	24,7-35,3	29,1-39,7	4,40	2,97	<,001*

Teste estatístico Generalized Mixed Model (GMM)

* diferença média é significativa no nível ,05

a Variável dependente: Leitura Acertos (Total= % de acerto escore total);

b Diferença Média

**Legenda:** AF= palavra de alta frequência, BF= palavra de baixa frequência; PP= pseudopalavra - teste PROLEC, pontuação padrão); Leitura Erros (Total= % de erro escore total). Texto narrativo (NAR); Texto expositivo (EXP); PPM= palavras por minuto; CO= compreensão de texto: % de questões corretas. Escrita Acertos Total, Palavras (Pal), Pseudopalavra (PP): valores referentes a média de % de acertos; Escrita Erros: Total: média % de erros. Pal.e,PP - % da pontuação padrão

### Monitoramento do desempenho

Ocorreu efeito significativo do tempo sobre a taxa de acertos totais na leitura (F=4,47/p=,003), especialmente entre as primeiras três listas (L1, L2, L3); em pseudopalavras (F=5,55/p=,001) e no índice de acurácia (F=2,73/p=,035). Não ocorreu mudança significativa na leitura de palavras de alta (F=,508/p>,05) e baixa frequência (F=1,49/p>,05). Ocorreu diminuição na taxa de erros totais (F=7,68/p<,001) entre as duas primeiras listas em comparação com as demais; em palavras de alta frequência (F=2,66/p=,040), baixa frequência (F=4,95/p=,002) e em pseudopalavras (F=10,20/p<,001). Na escrita não ocorreu aumento na taxa de acertos totais*,* mas quando os estímulos foram analisados separadamente, identificamos mudanças na escrita de palavras de baixa frequência (F=8,19/p<,001); de alta frequência (F=3,76/p=,008) e em pseudopalavras (F=3,49/p=,012). A diminuição da taxa de erros totais (F=2,87/p=,032) na comparação da L1 com a L5 (p=,012) pode reforçar os ganhos obtidos na pós avaliação, em que se notou aumento na pontuação do teste (indicativo de diminuição de erros). Análise dos estímulos indicou diferença significativa na taxa de erros para palavras de baixa frequência (F=3,19/p=,024), alta frequência (F=11,02/p<,001) e pseudopalavras (F=7,75/p<,001).

A análise da tipologia de erros para a leitura nos sete tempos (A1, A2 e cinco listas do monitoramento) indicou diminuição de erros por substituição de fonemas surdo-sonoros (F=13,13/p<,001); omissão e/ou acréscimo de letras (F=3,28/p=,006); desconhecimento das regras ortográficas (F=14,05/p<,001); troca de vogal (F=5,99/p<,001); lexicalização (F=5,96/p<,001); outros (F=2,56/p=,022). Na escrita, ocorreu diminuição de erros por trocas surdo-sonora (F=6,85/p<,001); inversão (F=3,15/p=,007); omissão e/ou acréscimo (F=6,63/p<,001); ortografia (F=20,35/p<,001); troca de vogal (F=6,63/p<,001); e outros (F=4,11/p<,001).

### Caracterização do perfil do Grupo de risco para Dislexia (GD) e do Grupo Não-dislexia (GND)

O Quadro 1 expõe a associação de critérios de análise qualitativa estabelecidos para a identificação do GD. O quadro expõe a análise retrospectiva das sessões de intervenção baseada nos registros de observação individual e corroboraram os resultados quantitativos obtidos nos testes estatísticos, visto que nenhum critério único é suficiente para configurar o perfil de dislexia.

**Quadro 1:** Critérios para identificação dos grupos perfil em risco para Dislexia vs Não-dislexia

**Avaliação Pré-Pós e Monitoramento**	**Dislexia**	**Não-dislexia**
Consciência fonológica	< taxa de variação, de ganho em pontuação	> taxa de variação, de ganho em pontuação
Acesso Lexical	< taxa de variação, de ganho em pontuação	> taxa de variação, de ganho em pontuação
Taxa de erros e acertos	Curva de aprendizagem oscilante ou estável, com pouca mudança	Diminuição gradativa de erros
	< taxa de variação, de ganho em pontuação	Aumento de acertos
		> taxa de variação, de ganho em pontuação
Tipologia de erros	Padrão específico na leitura e na escrita. Manutenção de erros por substituição dos pares fonêmicos surdo-sonoro.	Padrão variado
Velocidade de leitura	Evolução Lenta, baixa mudança	Variado
**Critérios nas sessões**	**Dislexia**	**Não-dislexia**
Consciência fonológica	Manutenção de dificuldade (ou avanços e retrocessos) em tarefas de: manipulação de palavras na frase (identificação, substituição de palavra); manipulação com sílabas; separação e junção de palavras em frases orais; manipulação de rimas, identificação e manipulação de fonemas.	Dificuldade nas primeiras sessões e diminuição de erros e/ou de dificuldades na execução das tarefas mais evidenciadas a partir da 6ª sessão.
Estimulação fonovisuoarticulatória	Manutenção de dificuldade nas atividades orais de discriminação dos pares fonêmicos; necessidade de múltiplas repetições; lentidão para automatizar estratégias de discriminação dos sons.	Dificuldade nas primeiras sessões, com diminuição gradativa; maior compreensão e identificação das diferenças dos pares; processo mais rápido de discriminação e manutenção de estratégias.
Leitura e escrita	Manutenção de erros por trocas surdo-sonoras mais evidenciadas após a 6ª sessão; lenta evolução na capacidade de autocorreção, necessidade de direcionamento constante.	Diminuição de erros por trocas surdo-sonoras mais evidenciadas a partir da 6ª sessão; evolução na capacidade de se autocorrigir; retomada espontânea de estratégias de discriminação ensinadas.
	Evolução lenta na correspondência fonema-grafema (sequencialização e soletração).	Manutenção de erros ortográficos, por regularização e contextuais.
	Manutenção de erros por hipo/hipersegmentação, pouca autocorreção.	
	Mantém erros ortográficos, por regularização e contextuais.	
Aspectos gerais (memória e acesso)	Lentidão em nomeação de figuras, com frequência se refere a “coisa”, “negócio” para nomear imagens mesmo que de categorias comuns (objetos do cotidiano, alimento, animais)	Padrão variado.
	Manutenção de dificuldades na manipulação mental.	Não foi observada dificuldade ou lentidão para nomeação de figuras, exceto pelo repertório de vocabulário ser reduzido.
		Manutenção de dificuldades na manipulação mental.

A análise da curva individual de erros/acertos na leitura/escrita identificou padrões que também foram analisados para discriminação dos grupos. A Figura 2 exemplifica o desempenho de dois participantes nas tarefas de leitura/escrita. A inspeção dos dados de comparação entre A1 e A2 evidenciou que participantes com desempenho abaixo de 25% na taxa de variação individual foram considerados baixo respondentes, comparados com os demais do grupo.

**Figura 2:** Perfil de mudança e comparação entre grupos

Eixo y: porcentagem de erros e/ou acertos em tarefa de leitura e escrita. Eixo x: Av. pré intervenção, Monitoramento Lista 1, Lista 2, Lista 3, Lista 4, Lista 5, Avaliação pós intervenção. Medidas avaliadas: LE (erros em leitura); EE (erros em escrita); LA (acertos em leitura); EA (acertos em escrita). P15-paciente do GD. P2-paciente GND

A associação dos critérios indicou 12 crianças com perfil de risco para dislexia. A análise de comparação evidenciou efeito significativo do grupo sobre a taxa de variação em consciência fonológica (Wald=6,074; p=,014); erros na leitura (Wald=4,103; p=,040) e escrita (Wald=4,367; p=,026); velocidade de leitura de texto expositivo (Wald=4,572; p=,032). O GD apresentou menores valores em média quanto ao ganho atingido nessas medidas em comparação com o GND (Tabela 3). Quanto à tipologia de erros na leitura*,* o GD cometeu mais erros por trocas surdo-sonoras (F=4,66; p=,040), enquanto o GND por desconhecimento de regras ortográficas (F=5,72; p=,021). A comparação entre médias e frequência indicou que embora o GD tenha cometido mais erros por lexicalização e por trocas de vogais, a diferença não foi confiável em p<0,05. Na escrita, o GD cometeu mais erros por troca surdo-sonora (F=9,52/p=,019), enquanto o GND por omissão e/ou acréscimo de letras (F=4,08/ p=,040). A comparação entre médias indicou que os erros por troca de vogais foram mais cometidos no GD, mas a diferença não foi confiável em p<0,05.

**Tabela 3:** Comparação das variações individuais por grupo

**Domínio**	**HD**	**M (DP)**	**Intervalo de confiança 95%**		B*	**Teste de hipótese**	
			**Inferior**	**Superior**		**Qui-Quadrado Wald**	**Sig.**
CFT	GD	11,63 (10,0)	-8,00	31,28	-33,60	6,07	,014*
	GND	45,24 (7,9)	29,64	60,83			
RAN (Letra)	GD	8,10 (7,0)	-5,79	22,00	-8,81	,83	,361
	GND	16,92 (5,6)	5,88	27,95			
RAN (Obj.)	GD	11,14 (6,0)	-,69	22,97	3,06	,13	,709
	GND	8,08 (4,7)	-1,31	17,47			
RAN (Num)	GD	6,11 (4,3)	-2,41	14,63	-1,16	,04	,836
	GND	7,27 (3,5)	,308	14,23			
MOV	GD	-,39(10,8)	-21,57	20,78	-14,21	1,03	,308
	GND	13,82 (8,8)	-3,46	31,11			
MOP	GD	6,06 (4,3)	-2,50	14,63	2,98	,266	,606
	GND	3,08 (3,78)	-4,33	10,50			
MOC	GD	7,60 (8,1)	-8,30	23,51	-8,44	,586	,444
	GND	16,05 (6,4)	3,42	28,67			
Leitura ER	GD	38,46 (6,5)	25,56	51,36	-17,44	4,21	,040*
	GND	55,91 (5,3)	45,38	66,44			
Leitura AC	GD	10,62 (4,4)	10,82	19,42	-10,51	2,96	,085
	GND	21,13 (3,5)	14,15	28,11			
Escrita ER	GD	23,38 (5,7)	12,13	34,62	-16,48	4,95	,026*
	GND	39,86 (4,6)	30,68	49,04			
Escrita AC	GD	9,56 (3,0)	3,67	15,44	-3,01	,54	,461
	GND	12,57 (2,3)	7,89	17,24			
PPM NAR	GD	15,38 (14,0)	-12,08	42,84	-26,25	1,79	,180
	GND	41,63 (12,3)	17,33	65,93			
PPM EXP	GD	13,87 (12,1)	-9,87	37,62	-35,90	4,57	,032*
	GND	49,77 (10,8)	28,42	71,12			

* Diferença entre a média da taxa de variação GD x GND. GR de referência: GD

Diferença significativa em p<0,05

**Legenda:** variáveis: CFT (Consciência fonológica total); Acesso lexical (nomeação automática rápida (RAN) de letras, objetos, números); MOV (memória operacional verbal dígitos); MOP (memória operacional palavras); MOC (memória operacional visual corsi). Leitura ER (erro); Leitura AC (acerto); Escrita ER(erro); Escrita AC (acerto); PPM NAR (velocidade de leitura palavra por minuto texto narrativo); PPM EXP (velocidade de leitura palavra por minuto texto expositivo). As covariáveis do modelo são fixadas nos seguintes valores: IDADE=9,27

## DISCUSSÃO

Este estudo desenvolveu e analisou resultados de um programa de detecção de sinais de risco para dislexia envolvendo estratégias interventivas com foco em habilidades cognitivo-linguísticas e treino de correspondência grafo-fonêmicas, como proposto na Camada 2 do modelo de Resposta à Intervenção realizado em contexto escolar. Para tanto, uma amostra de 30 crianças do Ensino Fundamental I foi submetida às atividades de intervenção, com monitoramento do desempenho e avaliação pré e pós intervenção.

Na avaliação pós-intervenção, analisamos inicialmente mudanças de desempenho na amostra como um todo e em seguida as funções cognitivo-linguísticas significativas para diferenciação entre perfil de risco para dislexia e dificuldade de aprendizagem. Mudanças estatisticamente significantes foram observadas em medidas de consciência fonológica, velocidade de acesso lexical, precisão na decodificação e codificação (taxa de acertos, taxa e tipologia de erros na leitura e na escrita), velocidade de leitura e compreensão textual. Mostraram-se significativas para a diferenciação dos perfis de desempenho os ganhos obtidos em consciência fonológica, aumento na velocidade de leitura textual e precisão de leitura e de escrita, expressa em diminuição da taxa de erros e tipologia de erros.

A memória operacional foi a única função em que não foram observadas mudanças na avaliação pós-intervenção. Já é bem conhecido que dificuldades na consolidação da leitura estão associadas a um curto *span* de dígitos^(22)^, sendo a alça fonológica do modelo de Baddeley^(23)^ responsável por permitir que itens verbais sejam mantidos mentalmente durante a manipulação necessária para o desempenho e emissão de respostas, como observado em tarefas de julgamento de rimas e repetição de pseudopalavras. No estudo de Maehler^(24)^ também não foram observadas mudanças de desempenho em tarefas de memória operacional verbal após um programa de intervenção com foco em leitura. O autor atribuiu tais resultados a uma baixa sensibilidade dos treinos sobre habilidades de memória, refletindo que estratégias mais ecológicas, que contemplem o funcionamento cotidiano da criança, poderiam ser mais eficazes.

Nossos achados revelaram escores mais altos na tarefa de nomeação automática rápida-RAN após a intervenção, o que indica aumento na velocidade de acesso lexical na amostra como um todo. Entretanto, os participantes com perfil de risco para dislexia não diferiram significativamente dos demais, o que sugere que tal habilidade não discriminou os grupos. Evidências prévias, entretanto, indicam que crianças disléxicas são, comumente, mais lentas na velocidade de acesso lexical^(25)^. Uma possível explicação para nossos achados é que tarefas de nomeação automática rápida demandam a integração de diversos recursos cognitivos, como atenção e velocidade de processamento. Assim, a variabilidade do desempenho pode ter sido influenciada por fragilidades cognitivas distintas da habilidade de nomeação.

As mudanças nas medidas de consciência fonológica e das habilidades de decodificação/codificação mostraram-se especialmente importantes na identificação de crianças de risco para dislexia. Por exemplo, observou-se um ganho no desempenho na tarefa de consciência fonológica de cerca de 33% a mais entre os participantes considerados como sem risco. Um desenvolvimento desta habilidade em crianças com dificuldades em leitura e escrita submetidas a programas de estimulação fonológica e multissensorial tem sido amplamente relatado^(26,27)^ .

Na tarefa de leitura, nossos achados revelaram melhora na acurácia de decodificação independentemente do nível de frequência (alta e baixa) e lexicalidade (palavras e pseudopalavras), além de diminuição da taxa de erros na etapa pós intervenção da amostra como um todo. Nas tarefas de monitoramento, entretanto, houve ganhos somente na leitura de pseudopalavras. Tilanus et al.^(28)^ também observaram melhor precisão de leitura de pseudopalavras em comparação a palavras regulares em crianças com perfil de risco para dislexia após uma intervenção de 12 semanas com ênfase em treino de correspondência grafema-fonema. Em nosso estudo, apesar do progresso constante observado em pseudopalavras, ainda verificamos maiores dificuldades na decodificação destes estímulos em comparação com as palavras de alta frequência. Diferenças na acurácia de leitura de palavras e pseudopalavras são esperadas dado que palavras de alta frequência são habitualmente lidas com mais facilidade. Quanto mais frequente a exposição a uma palavra, maior a possibilidade de armazenamento no léxico semântico e ortográfico, levando prioritariamente à leitura pela rota lexical. Esse processo não ocorre com as pseudopalavras, já que demandam acesso ao léxico fonológico^(6,8)^. Ao compararmos os grupos, constatamos que os não-disléxicos apresentaram 17,44% a mais na diminuição de erros pós-intervenção, em comparação com os demais. De fato, os participantes com perfil de risco para dislexia não apresentaram mudanças substanciais na quantidade de erros cometidos. Além disso, observou-se importante diferença entre precisão e velocidade de leitura nas avaliações de monitoramento. Embora não tenha sido observado um aumento substancial no número de palavras lidas corretamente, os participantes mostraram maior velocidade de leitura apesar de em níveis ainda abaixo do esperado para a faixa etária e escolarização. Esse achado pode ser justificado pelo grau de dificuldades identificadas no início da intervenção e também pelo déficit nas habilidades fonológicas^(5)^ que dificultam a decodificação. Considerando que a rota lexical é essencial para a fluência de leitura, uma vez que favorece a compreensão textual^(5,6)^, era esperado que com o baixo desempenho na fluência, a compreensão de texto seria igualmente difícil. Tanto a velocidade como a compreensão demandam processos cognitivo-linguísticos bem desenvolvidos, incluindo componentes sintáticos, semânticos, inferenciais e integração na memória.

A melhora na escrita de palavras foi evidenciada mais em redução de erros do que em aumento de acertos. Ou seja, os participantes passaram a errar menos nas correspondências fonema-grafema, uma melhora mais expressiva do que o aumento de acertos na palavra global. A frequência de erros após a intervenção foi de 16,48% a menos entre os participantes sem perfil em risco de dislexia. Efeitos eletivos de intervenção em instrução fônica em termos de redução na taxa de erros de decodificação tanto em palavras quanto em pseudopalavras também foram relatados em estudos internacionais^(28)^. Estudos brasileiros^(29,30)^ envolvendo crianças de ensino fundamental em geral relataram apenas aumento de acertos. Em nosso estudo, constatamos que explorar tipos de erros pode oferecer indicadores da estratégia utilizada pela criança na leitura e na escrita. Por exemplo, palavras irregulares só podem ser acessadas pela rota lexical, já que a correspondência é arbitrária e a criança deve memorizar como a palavra é pronunciada^(6)^. Quando o estoque de palavras armazenadas no léxico ortográfico é reduzido, como em decorrência de menor exposição à leitura, podem ocorrer erros por regularização da língua e/ou contextuais. Erros desta natureza foram observados com maior frequência entre as crianças com dificuldades de aprendizagem após a intervenção. Participantes com perfil de risco para dislexia cometeram mais erros por substituição dos pares fonêmicos surdo-sonoros, tanto na leitura quanto na escrita, podendo indicar prejuízos na rota fonológica, importante para a precisão na conversão grafofonêmica. Esses achados foram semelhantes a outros estudos que compararam os tipos de erros na leitura e escrita entre amostras de disléxicos e não-disléxicos^(29,30)^.

Conforme o modelo tradicional de RTI^(2,3)^, assim que uma criança de risco é identificada sua capacidade de resposta à intervenção deve ser monitorada. Nosso estudo utilizou o monitoramento como um processo de avaliação sistemática e contínua de desempenho em leitura e escrita, para compreender a curva de aprendizagem. Diferenças no perfil dessa curva foram um dos parâmetros para discriminar crianças com perfil de risco de dislexia daquelas com dificuldades de aprendizagem. A aplicação de instrumentos padronizados de investigação de habilidades cognitivo-linguísticas antes e após a intervenção também gerou medidas de ganhos no desempenho. Outro aspecto importante é que uma única variável para medir a responsividade da criança mostrou-se pouco discriminativa, provavelmente pela aprendizagem ser um processo que engloba diferentes habilidades. Dessa forma, análises quali-quantitativas têm maior potencial para lidar com a heterogeneidade das dificuldades de aprendizagem.

A evolução nas habilidades fonológicas foi particularmente importante para a compreensão das dificuldades de leitura e escrita. Participantes com perfil de risco para dislexia, embora tenham obtido melhoras, demonstraram maior esforço ao longo das sessões e necessitaram de mais tempo até automatizarem estratégias de correspondências grafo-fonêmicas, sendo considerados baixo respondentes. Estudos brasileiros^(3)^ e internacionais^(26)^ baseados no modelo tradicional de RTI também abordam as habilidades fonológicas e de decodificação como medidas importantes para discriminar crianças respondentes ou baixo respondentes. As observações do presente estudo sugerem ainda que o perfil de risco para dislexia esteja associado a uma baixa responsividade, tal como evidenciada em uma taxa (mínima) estabelecida de variação menor que 25%, no que concerne às habilidades linguísticas e aos erros de decodificação. O perfil parece ainda associado a curvas de aprendizagem oscilantes, identificadas por meio do monitoramento do desempenho. Isto é, a taxa de ganho atingido na intervenção, ou seja quanto o desempenho mudou após a intervenção na comparação com o resultado inicial, foi menor entre as crianças identificadas com perfil de risco para dislexia. Reforça-se que esses dados não indicam que elas não melhoraram, mas que apresentaram evoluções mais lentas. Estudos longitudinais também evidenciaram que crianças disléxicas tendem a permanecer com padrão de leitura e escrita lento e não-automático^(5,31)^. O DSM-5^(1)^ também descreve esse perfil de evolução em crianças com dislexia na medida em que pontua a baixa precisão de resposta como um dos critérios diagnósticos. Isso ocorre porque a dificuldade é persistente e o alcance de desempenhos, ainda que medianos, demanda grande esforço e mediação constante. Esse padrão configura uma discrepância entre o que é esperado em termos de resposta a intervenções em leitura e escrita e o que a criança de fato conseguiu evoluir, aproximando-se dos pressupostos do RTI.

Nossos achados reforçam os relatos prévios de que as expressões cognitivas da dislexia são heterogêneas, mas as dificuldades de aprendizagem também podem variar substancialmente em termos de manifestações bem como de causas. Os participantes sem sinais críticos de dislexia apresentaram algumas características de desempenho semelhantes (mas não específicas) às das demais, porém de etiologia distinta. Identificamos que em 60% dos casos as dificuldades foram justificadas por questões comportamentais, psicopedagógicas, necessidade de adaptações aos contextos escolares, baixa motivação ou associadas a outros transtornos do neurodesenvolvimento. Identificamos ainda que 77,8% das crianças com dificuldades de aprendizagem eram provenientes de escolas públicas. Esses resultados podem refletir diferenças socioeconômicas e ambientais, incluindo as fragilidades do sistema educacional brasileiro. Sendo assim, reforça-se a necessidade da identificação e intervenção precoce em crianças que apresentem dificuldades de aprendizagem no ensino fundamental, visando minimizar as repercussões emocionais e comportamentais.

Nessa perspectiva, nossos achados indicam que a abordagem de resposta à intervenção constitua uma alternativa mais válida para a identificação de crianças de risco para dislexia do que a baseada em única avaliação do desempenho escolar e de funções cognitivas, evitando-se assim casos falso-positivos e/ou espera do diagnóstico. Nossos achados reforçam que um modelo de resposta à intervenção associado a processos diagnósticos deve seguir passos como triagem das crianças para identificação dos sintomas de risco para dislexia, intervenção em habilidades cognitivo-linguísticas, monitoramento do desempenho e reavaliação. Para discriminar crianças com dislexia por meio da análise da responsividade à intervenção, os seguintes procedimentos se mostraram facilitadores^(1)^: analisar mudanças em medidas de consciência fonológica e quantificá-las considerando parâmetros de ganho^(2)^; analisar o padrão da curva de aprendizagem em medidas de leitura e escrita, prioritariamente a taxa de erros^(3)^; identificar tipologia de erros, principalmente a persistência de substituição dos pares fonêmicos surdo-sonoros. O esquema de 12 sessões semanais de duas horas de duração e a configuração em grupo de cinco participantes mostraram-se viáveis para detecção de casos de risco para dislexia. A característica em sessões com caráter avaliativo e remediativo alinhadas a um monitoramento das mudanças de desempenho parece permitir maior segurança diagnóstica. Adicionalmente, reforça-se a importância da abordagem multiprofissional no trabalho com crianças de baixo rendimento acadêmico, na área da saúde e da educação.

Como limitações do estudo consideramos que os parâmetros (ponto de corte) utilizados para quantificar e qualificar a responsividade à intervenção podem não ser úteis em todos os contextos e para todas as crianças, em função da heterogeneidade das dificuldades de aprendizagem. Ou seja, embora pontos de corte em medidas quantitativas podem contribuir para discriminar bons e maus leitores, eles ainda são arbitrários. Sabe-se que as habilidades acadêmicas se distribuem ao longo de um *continuum* que abrange a capacidade e a incapacidade de ler, um conceito chamado de modelo dimensional^(1)^. Entendemos que o programa diagnóstico-interventivo proposto no presente estudo se aproxima mais de uma perspectiva diagnóstica dimensional do que categórica baseada na ideia da descontinuidade, ou seja, uma ruptura entre um bom leitor e um “leitor deficiente”^(1)^. Pesquisas futuras poderão propor outros critérios de discriminação, visto a necessidade de aprimoramento de processos diagnósticos.

## CONCLUSÃO

Este estudo desenvolveu e analisou a adequação de um programa de intervenção em dificuldades de leitura e escrita baseado no modelo de Resposta à Intervenção. O programa envolveu estimulação de habilidades fonológicas e ensino sistemático de correspondências grafofonêmicas, monitoramento das aquisições e avaliação pré e pós intervenção. Habilidades de consciência fonológica, velocidade de leitura e frequência e tipos de erros em leitura e escrita foram as que se mostraram mais adequadas na identificação das 12 crianças com perfil de risco para dislexia. Programas desta natureza podem favorecer a interlocução entre profissionais de saúde e de educação para o atendimento a estudantes do ensino fundamental bem como evitar risco de falsos positivos em dislexia no contexto clínico. Mais pesquisas são necessárias para analisar modelos de resposta à intervenção voltados para um diagnóstico assertivo da dislexia e, especialmente, sobre a aplicabilidade do modelo RTI no Brasil.
